# APOE Variant (rs405509) might Modulate the Effect of Sex and Educational Level on Cognitive Impairment Risk in a Taiwanese Population

**DOI:** 10.3390/ijerph16101732

**Published:** 2019-05-16

**Authors:** Tsui-Wen Hsu, Disline Manli Tantoh, Pang-Li Liu, Pei-Hsin Chen, Oswald Ndi Nfor, Ming-Chih Chou, Long-Yau Lin, Yung-Po Liaw

**Affiliations:** 1 Institute of Medicine, Chung Shan Medical University, Taichung 40201, Taiwan; irb@cgh.org.tw; 2 Superintendent Office, Cathay General Hospital, Taipei 106, Taiwan; 3 Department of Public Health and Institute of Public Health, Chung Shan Medical University, Taichung 40201, Taiwan; tantohdisline@yahoo.com (D.M.T.); c0701chen@gmail.com (P.-H.C.); nforoswald2@yahoo.com (O.N.N.); 4 Department of Medical Sociology and Social Work, Chung Shan Medical University, Taichung 40201, Taiwan; lpl@csmu.edu.tw; 5 School of Medicine, Chung Shan Medical University, Taichung 40201, Taiwan; 6 Department of Family and Community Medicine, Chung Shan Medical University Hospital, Taichung 40201, Taiwan

**Keywords:** education, sex, MMSE, APOE, rs405509

## Abstract

Education, sex, and the APOE-rs405509 variant are associated with Alzheimer’s disease and cognitive performance. We investigated if the rs405509 TT, TG, and GG genotypes modulate the effect of sex and education on cognitive impairment in Taiwanese adults. Data on cognitive health (defined by Mini-Mental State Examination (MMSE) scores) and rs405509 were from Taiwan Biobank. Participants included 2105 men and 2027 women with a mean age of 64 years. Education below university level was significantly associated with lower MMSE scores. The odds ratios (ORs) were 1.82; 95% confidence interval (CI) 1.38–2.41 for senior high school, 3.39; 95% CI 2.50–4.59 for junior high school, and 11.94; 95% CI 9.91–15.50 for elementary school and below (*p*-trend < 0.05). The association between MMSE score and sex was significant only in the lowest educational group (elementary and below), with lower odds of having a low MMSE score in men compared to women (OR = 0.51; 95% CI 0.34–0.77). After stratification by rs405509 genotypes, this association was significant only among TT genotype carriers (OR = 0.481; CI = 0.253–0.915). In conclusion, a significant association between MMSE score and sex was observed in the lowest educational group, especially among carriers of rs405509 TT genotypes.

## 1. Introduction

Mini-Mental State Examination (MMSE) is one of the commonly used cognitive function screening scales [1]. It has been broadly used to screen for dementia in people with suspected cognitive impairments [2,3]. The MMSE has a total score ranging from 0 to 30, with lower scores being an indicator for worse cognitive impairment. [4] A score of 24 has been established as the cut-off point [5].

People with mild cognitive impairment (MCI) have been associated with a higher risk of dementia [6]. The number of people with dementia worldwide is expected to be 75.63 million in 2030 and 135.46 million in 2050 [7]. Taiwan is one of three countries in Asia where approximately 9.5 million people are currently living with dementia [8]. Early identification of individuals with cognitive impairment might help to reduce the risk of developing dementia and Alzheimer’s disease.

Age, educational level, gender, and genetic factors are some of the potential variables that have been associated with cognitive abilities [9,10]. Younger adults have presented higher MMSE scores (indicating higher cognitive performance) [3]. In Taiwan, female sex and lower educational levels have been associated with cognitive impairment [11]. In other populations, higher education has been associated with a better cognitive performance [12]. The educational level of a parent has an impact on the offspring’s intelligence. Educational attainment is among the variables believed to serve as protective factors in dementia [13]. The APOE gene influences the human brain and is one of the significant genetic factors associated with the development of Alzheimer’s disease, which is also associated with a cognitive performance [14,15,16,17]. In particular, the T allele of the rs405509 single nucleotide polymorphism (SNP) located on the promoter of the APOE gene is a well-established variant associated with Alzheimer’s disease and cognitive performance [14,15,16,17].

As stated above, cognitive function is affected by several independent variables including sex, education and genetic factors such as the rs405509 variant. According to findings from a previous study, sex alone did not affect MMSE scores, whereas the interaction of education and sex showed significant effects [3]. The impact of the aforementioned variables on MMSE scores has been demonstrated in other populations. However, such investigations have not been fully explored in Taiwan. Therefore, using Taiwan Biobank participants, we investigated the independent effect of sex, age, educational level, and their interaction on MMSE among the elderly population in Taiwan.

## 2. Methods

### 2.1. Data Source

Data were obtained from the Taiwan Biobank, a national health resource that is open to researchers. It contains health information on ethnic Taiwanese residents (aged 30–70 years) from 2008–2016 [18,19,20]. The biobank aims to facilitate the development of better prevention and treatment strategies for chronic disease like cancer, heart disease, cerebrovascular disease, diabetes, chronic hepatitis, liver cirrhosis, hypertension and other illness factors that are among the ten leading causes of death in Taiwan. Currently, it contains health data from approximately 109,747 residents collected from 29 recruitment centers distributed across the country. Available data are separated into five categories including questionnaires, physical examination, blood and urine tests, biological samples, and experimental data (whole-genome genotyping, whole-genome sequencing, DNA methylation, human leukocyte antigen (HLA) typing, and blood metabolome). Recruitment of individuals in the Taiwan Biobank project conforms to relevant regulations and guidelines. Written informed consent was obtained from each participant prior to data collection. The Institutional Review Board of Chung Shan Medical University (CS2-16114) approved this study.

### 2.2. Study Participants and Cognitive Assessment

Data on the cognitive health of 4132 adults (2027 women and 2105 men) aged 60–70 years were obtained from questionnaires contained in Taiwan Biobank. The biobank participants were administered the Mini-Mental State Examination that included tests of orientation, attention, memory, language and visual-spatial skills. The main outcome of the study was MMSE. Participants’ score ranged from 0 to 30 points, with a cut-off of 24 [21]. Variables examined included APOE-rs405509 genotypes (TT, TG, and GG), age, gender, high-density lipoprotein (HDL-C), body mass index (BMI), educational levels, marital status, smoking, and drinking habits. Participants were classified based on their educational levels. Information on the school grades completed by the participants was registered, and the levels (from higher to lower) included (1) university and above, (2) senior high school, (3) junior high school, and (4) elementary school and below.

### 2.3. Genetic Variant Selection and Genotyping

Through a literature search (Pub Med, ScienceDirect, Google Scholar, SNPedia, and GWAS Catalog), we identified rs405509, a well-established variant in APOE gene that has been previously associated with Alzheimer’s disease and cognitive performance. SNP genotyping was carried out using the custom Taiwan Biobank chips and run on the Axiom™ Genome-Wide Array Plate System (Affymetrix, Santa Clara, CA, USA).

### 2.4. Statistical Analysis

Data management and statistical analyses were performed using SAS 9.3 software (SAS Institute, Cary, NC, USA). Associations of the sex with categorical variables were assessed using the Chi-square test. Student’s T-test was used to compare the differences in continuous variables between men and women. Multivariate logistic regression models were used to determine associations with MMSE scores. Adjustments were made for covariates including age, smoking, hypertension, diabetes, BMI, alcohol intake, waist-hip ratio (WHR), marital status, and physical activity. Data were presented as means ± S.E (continuous variables) or numbers (%). PLINK 1.09 beta was used to analyze genotypic data. The Hardy–Weinberg equilibrium (HWE) test was performed for the rs405509 SNP. We excluded SNPs if the minor allele frequency (MAF) was <0.05. We also excluded SNPs whose genotypes deviated from the Hardy–Weinberg equilibrium (HWE). *p*-values < 0.05 were considered to be statistically significant.

## 3. Results

Demographic variables of the study participants are shown in Table 1. Participants in the study included 2105 men and 2027 women with a mean age ± SE of 64.04 ± 0.06 and 64.04 ± 0.07, respectively. Education level completed by the participants ranged from university and above to elementary and below. Associations of MMSE scores with educational levels are shown in Table 2. Participants who had university education below university level were significantly associated with lower MMSE scores. The odds ratios (ORs) were 1.82 (95% confidence interval [CI] 1.38–2.41) for senior high school, 3.39 (95% CI 2.50–4.59) for junior high school, and 11.94 (95% CI 9.91–15.50) for elementary school and below. The test for trend was statistically significant. The higher the educational level, the higher the MMSE scores. Age was also associated with lower MMSE scores (OR = 1.08, CI = 1.05–1.12). The interaction between educational level and sex was significant (*p* = 0.022). After stratification by sex, significant associations were shown between education below university level and lower MMSE scores, with higher odds ratios among female than male participants (Table 3). The OR in men was 1.95 (95% CI 1.34–2.82) for senior high school, 2.92 (95% CI 1.86–4.60) for junior high school, and 8.99 (95% CI 6.23–12.98) for elementary school and below. Likewise, those for women were 1.85 (95% CI 1.20–2.85) for senior high school, 4.00 (95% CI 2.57–6.21) for junior high school, and 15.20 (95% CI 1.02–1.11) for elementary school and below. Table 4 shows the association between sex and MMSE score by different educational levels. The association between the MMSE score and sex was only significant in the lowest educational group (elementary and below) and the odds of having a low MMSE score was lower in men compare to women OR = 0.51 (95% CI = 0.34–0.77), indicating that men had a significantly lower risk for cognitive impairment than women. After stratification by rs405509 genotypes, the association between MMSE score and sex was significant only among TT carriers in the lowest educational group (OR = 0.445, CI = 0.236–0.838) as shown in Table 5. Further stratification by covariates (Table 6) suggested that significant associations remained among participants in the lowest educational group and specifically among those with no history of hypertension (OR = 0.519, CI = 0.310–0.868 or diabetes (OR = 0.487, CI = 0.300–0.788), as well as those with abnormal WHR (OR = 0.584, CI = 0.359–0.950).

## 4. Discussion

This study was undertaken to assess the mini-mental state examination score in Taiwanese adults, emphasizing the impact of sex and educational level and their interaction in individuals with the APOE rs405509 variant. MMSE was chosen because of its wide use in clinical and research settings to screen for the presence or severity of dementia. In general, education below university level, and age were significantly associated with a lower MMSE score, indicating a higher risk for cognitive impairment. After stratification by sex, education below university level and age remained significantly associated with a higher risk of cognitive impairment in women compared to men. We also found that the impact of the interaction between sex and educational level on the MMSE score was significant. However, after stratification by genotypes in APOE rs405509 variant, the sex effect on MMSE score was significant only among TT carriers in the lowest educational level (elementary school and below). Further stratification by covariates suggested that significant associations remained in the lowest educational group and specifically among participants with no history of hypertension or diabetes, as well as those with abnormal WHR.

In line with our study, a significant interaction between sex and educational level on MMSE score was demonstrated in a previous study. When sex, educational level, and age were considered independently, only educational level and age were significantly associated with the MMSE score [3]. In addition, several other previous studies showed similar associations between MMSE score and educational level [2,22,23,24,25,26], age [2,25,26,27,28], and sex [2,24]. However, no significant associations of MMSE scores with educational level [23,27,29] and age have been reported [24]. Furthermore, MMSE scores were significantly attributed to sex differences [30,31]. However, interactions between educational level and sex were not significant [31].

Several previous studies have shown that poorer cognitive performance is independently associated with the female sex [26,28,32]. In some previous studies, the differences in total MMSE scores between men and women were significant only among individuals whose educational levels were low with men having better scores than women [31,33]. Similarly, in our study, the association between MMSE score and sex was only significant in the lowest educational group (elementary and below) and the scores were relatively higher in men than women. Unlike our findings, women have been reported to have better cognitive function than men, regardless of their lower educational level [34]. The APOE gene is regarded as a biomarker with the highest known impact on cognitive function [35]. It has been suggested to play this cognitive role from early life by influencing the educational level that one could attain [36]. The T allele of the rs405509 polymorphism has been shown to be a modulator of APOE’s effect on cognitive performance [17]. In our study, the sex effect on MMSE score was significant only among TT carriers in the lowest educational level (elementary school and below).

The mechanism behind the influence of age and education on MMSE score which mirrors cognitive performance is yet to be fully elucidated. The functional connectivity of the brain [37,38] alongside cognitive [39,40] and brain reserve [41] theories are some the mechanisms of actions that have been proposed. For instance, the brain’s functional connectivity has positive and negative associations with education [37] and age [37,38], respectively. These patterns conform with the cognitive reserve theory [39,40], and brain reserve theory [41]. The brain network of individuals with higher educational levels have better functional connections, which render them more efficient compared to those with lower levels [37,42].

MMSE score may have the ceiling effect. Francisco and colleagues found that poorly educated persons were more prevalent on the side of MMSE ceiling effects [43]. They concluded that even when MMSE scores are corrected for educational level, ceiling and floor effects are more likely to occur. However, in our study, we focused on the effect of different educational levels and gender on MMSE scores. Therefore, the ceiling effect on MMSE may be of minimal concern. It is possible that survival bias could be introduced across different educational levels. Such bias would be considered as a non-differential misclassification that can result in bias toward null.

## 5. Conclusions

In summary, a significant effect of the interaction between sex and educational level on the MMSE score was found in our study. MMSE score was significantly associated with sex in the lowest educational group (elementary and below). The odds of having a low MMSE score was lower in men compared to women, indicating a higher risk of cognitive impairment in women. However, after stratification by genotypes in the APOE rs405509 variant, we found that the sex effect on MMSE score was significant only among TT carriers in the lowest educational group.

## Figures and Tables

**Table 1:** Demographic characteristics of study participants.

Variable	Men	Women	*p*-Value
	*n* = 2105 (%)	*n* = 2027 (%)	
**MMSE**			0.0001
<24	255 (12.11)	414 (20.42)	
≥24	1850 (87.89)	1613 (79.58)	
**Education Level**			<0.0001
University and above	1075 (51.07)	583 (28.76)	
Senior High School	552 (26.22)	623 (30.74)	
Junior High School	212 (10.07)	333 (16.43)	
Elementary and below	266 (12.64)	488 (24.07)	
**Age (mean ± S.E.)**	64.04 ± 0.063	64.04 ± 0.066	0.928
**Marital Status**			<0.0001
No	178 (8.46)	642 (31.67)	
Yes	1927 (91.54)	1385 (68.33)	
**Drinking**			<0.0001
Never	1710 (81.24)	1997 (98.52)	
Former	155 (7.36)	11 (0.54)	
Current	240 (11.40)	19 (0.94)	
**Smoking**			<0.0001
Never	1172 (55.68)	1980 (97.68)	
Former	680 (32.30)	25 (1.23)	
Current	253 (12.02)	22 (1.09)	
**HDL (mean ± S.E.)**	48.57 ± 0.250	57.72 ± 0.295	<0.0001
**BMI (mean ± S.E.)**	24.68 ± 0.064	24.06 ± 0.074	<0.0001
**Physical Activity**			0.040
No	687 (32.64)	723 (35.67)	
Yes	1418 (67.36)	1304 (64.33)	

MMSE = mini mental state examination, S.E = standard error, HDL = high-density lipoproetein, BMI = body mass index.

**Table 2:** Multivariate logistic regression analysis showing associations with MMSE scores.

Variables	OR	95% CI	*p*-Value
**Sex**			
Women	Ref	-	-
Men	0.79	0.62–1.00	0.053
**Education Level**			
University and above	Ref	-	-
Senior High School	1.82	1.38–2.41	<0.0001
Junior High School	3.39	2.50–4.59	<0.0001
Elementary and below	11.94	9.19–15.50	<0.0001
**P for trend**			<0.0001
**Age**	1.08	1.05–1.12	<0.0001
**Marital status**			
No	Ref	-	-
Yes	1.10	0.88–1.39	0.396
**Drinking**			
Never	Ref	-	-
Former	1.02	0.61–1.73	0.930
Current	0.65	0.42–1.01	0.056
**Smoking**			
Never	Ref	-	-
Former	0.88	0.65–1.20	0.413
Current	1.10	0.73–1.66	0.644
**HDL**	1.00	0.99–1.01	0.467
**BMI**	1.02	0.99–1.05	0.307
**Physical activity**			
No	Ref	-	-
Yes	0.98	0.81–1.18	0.806

OR = odds ratio, CI = 95% confidence interval, BMI = body mass index, HDL-C = high-density lipoprotein-cholesterol. A higher value of OR in this table means a lower MMSE score.

**Table 3:** Multivariate logistic regression analysis showing associations with Mini-Mental State Examination (MMSE) scores stratified by sex.

Variables	Men			Women		
	OR	95% CI	*p*-Value	OR	95% CI	*p*-Value
**Education Level**						
University and above	Ref	-	-	Ref	-	-
Senior High School	1.95	1.34–2.82	0.000	1.85	1.20–2.85	0.006
Junior High School	2.92	1.86–4.60	<0.000	4.00	2.57–6.21	<0.0001
Elementary and below	8.99	6.23–12.98	<0.000	15.20	10.21–22.63	<0.0001
***p* for trend**	2.04	1.81–2.30	<0.000	2.64	2.34–2.97	<0.0001
**Age**	1.10	1.05–1.16	<0.000	1.07	1.02–1.11	0.003
**Marital Status**						
No	Ref	-	-	Ref	-	-
Yes	1.41	0.84–2.39	0.195	1.03	0.79–1.35	0.808
**Drinking**						
Never	Ref	-	-	Ref	-	-
Former	0.98	0.56–1.71	0.934	1.28	0.29–5.62	0.747
Current	0.70	0.44–1.12	0.136	0.46	0.12–1.71	0.245
**Smoking**						
Never	Ref	-	-	Ref	-	-
Former	0.91	0.66–1.25	0.543	0.81	0.28–2.38	0.703
Current	1.11	0.72–1.72	0.632	1.14	0.37–3.49	0.824
**HDL**	1.00	0.99–1.01	0.941	1.00	0.99–1.01	0.334
**BMI**	1.01	0.96–1.06	0.710	1.02	0.98–1.06	0.387
**Physical Activity**						
No	Ref	-	-	Ref	-	-
Yes	0.81	0.61–1.09	0.161	1.11	0.86–1.43	0.446
Educational Level * Sex						*p* = 0.022

*OR = odds ratio, CI = 95% confidence interval, BMI = body mass index, HDL-C = high-density lipoprotein-cholesterol. A higher value of OR in this table means a lower MMSE score.

**Table 4:** Effect of sex on MMSE scores with respect to educational level.

Education Level	*n*		OR	95% CI
	≤24	>24		
University and above	98	1560	1.02	0.61–1.71
Senior High School	125	1050	0.91	0.56–1.49
Junior High School	104	441	1.05	0.60–1.84
Elementary and below	342	412	0.51	0.34–0.77

Adjusted for age, marital status, smoking habit, alcohol drinking, BMI, HDL, physical activity, hypertension, diabetes, and WHR. Values of OR in this table indicate the odds of having a low MMSE score (≤24) in men compared to women.

**Table 5:** Effect of sex on MMSE score with respect to educational levels and genotypes.

Educational Level	TT		GT		GG	
	OR	95% CI	OR	95% CI	OR	95% CI
University and above	1.282	0.563–2.917	1.385	0.656–2.927	<0.001	<0.001–>999.999
Senior high school	1.461	0.682–3.130	0.842	0.421–1.685	0.556	0.044–7.030
Junior high school	1.466	0.668–3.214	0.760	0.291–1.990	<0.001	<0.001–>999.999
Elementary and below	0.481	0.253–0.915	0.639	0.323–1.261	0.757	0.174–3.298

Adjusted for age, marital status, drinking habit, smoking habit, HDL, BMI, and physical activity, hypertension, diabetes, and WHR. Values of OR in this table mean the odds of having a low MMSE score in men compared to women across different educational levels and genotypes.

**Table 6:** Effect of sex on MMSE scores with respect to educational level and risk factors.

	No Hypertension		Hypertension		No Diabetes		Diabetes		Normal WHR		Abnormal WHR	
	OR	95% CI	OR	95% CI	OR	95% CI	OR	95% CI	OR	95% CI	OR	95% CI
University and above	1.225	0.677–2.216	2.927	0.559–15.323	1.312	0.731–2.357	1.356	0.312–5.891	0.974	0.449–2.114	1.871	0.872–4.014
Senior high school	0.769	0.415–1.424	2.077	0.826–5.224	0.861	0.484–1.534	1.942	0.680–5.545	0.910	0.375–2.211	1.102	0.605–2.009
Junior high school	1.055	0.522–2.132	1.263	0.373–4.277	1.101	0.568–2.136	1.016	0.258–3.995	2.253	0.743–6.831	0.743	0.358–1.543
Elementary school and below	0.519	0.310–0.868	0.639	0.293–1.396	0.487	0.300–0.788	0.814	0.315–2.104	0.457	0.183–1.142	0.584	0.359–0.950

*Adjusted for age, genotypes, marital status, drinking habit, smoking habit, HDL, BMI, and physical activity. Values of OR in this table mean the odds of having a low MMSE score in men compared to women.
